# Role of *VOPP1* in regulation of Paclitaxel response and EMT process during ovarian tumor progression

**DOI:** 10.22099/mbrc.2025.52515.2107

**Published:** 2025

**Authors:** Mohammad Hossein Khajavirad, Amirhosein Maharati, Negin Taghehchian, Fatemeh Taghavinia, Meysam Moghbeli

**Affiliations:** 1 Student Research Committee, Faculty of Medicine, Mashhad University of Medical Sciences, Mashhad, Iran; 2 Medical Genetics Research Center, Mashhad University of Medical Sciences, Mashhad, Iran

**Keywords:** Ovarian cancer, Drug resistance, VOPP1, EMT, Tumor suppressor

## Abstract

Ovarian cancer (OC) is one of the most common malignancies of the genitourinary system in women that has a high mortality rate worldwide. Drug resistance and tumor relapse are the main causes of high mortality rate in OC patients. Therefore, investigation of the molecular mechanisms involved in OC progression can be valuable to introduce effective therapeutic targets for these patients. Epithelial-mesenchymal transition (EMT) as a key regulator of tumor relapse and drug resistance can be regulated by different signaling pathways such as WNT and NOTCH. *VOPP1* is an activator of NF-κB pathway during tumor progression. Considering the importance of cross talks between different signaling pathways during tumor progression, we assessed the role of *VOPP1* in OC progression through the modulation of WNT and NOTCH pathways. The expression levels of components of WNT and NOTCH signaling pathways, as well as the EMT process, were evaluated in *VOPP1*-induced A2780 cells compared to control cells. Role of *VOPP1* in OC invasiveness was also assessed through migration and drug resistance assays. *VOPP1* inhibited EMT process and NOTCH and WNT pathways in A2780 cells. *VOPP1* also significantly reduced cell migration (*p*=0.04) and paclitaxel (PTX) resistance in A2780 cells (*p*<0.0001). *VOPP1* reduced ovarian tumor cell migration and PTX resistance via regulation of NOTCH and WNT mediated EMT process. Therefore, it can be suggested as a novel therapeutic target in OC patients following further animal studies and clinical trials.

## INTRODUCTION

Ovarian cancer (OC) is a serious global health challenge, affecting 324,603 individuals and causing approximately 200,000 deaths annually [1]. Ovarian cancer is a heterogeneous disease, comprising various tumor types with distinct clinical characteristics and prognoses. They are classified based on their tissue of origin into epithelial cells, stromal endocrine cells, and totipotent germ cells [2]. The standard treatment regimen for advanced OC patients involves platinum-based chemotherapy combined with a taxane as adjuvant therapy [3]. However, up to 70% of patients with advanced-stage OC experience disease recurrence and metastasis despite these treatments. Since, retreatment with platinum agents is also ineffective for platinum-resistant patients, alternative therapeutic options are necessary for these cases [4, 5]. Therefore, Novel approaches, including targeted therapies and immunotherapy, are emerging as potent options to improve outcomes in these patients.

Epithelial-to-mesenchymal transition (EMT) is a biological process during which epithelial cells lose E-cadherin and acquire mesenchymal markers, including N-cadherin and vimentin that results in tumor metastasis [6, 7]. EMT-inducing transcription factors including Snail, Slug, *ZEB1*, *ZEB2*, and *TWIST1* not only repress the epithelial markers, but also up regulate mesenchymal markers and Matrix Metalloproteinases, which contribute to the disruption of epithelial cell adhesions and the promotion of EMT [8, 9]. EMT can be regulated by various signaling pathways such as NF-κB, Wnt/β-catenin, and Notch [10, 11]. Hypoxia and growth factors such as *HGF* also contribute to EMT by activation of PI3K/AKT and *Src* [12-14]. According to the vital role of EMT in tumor recurrence, metastasis, and drug-resistance [15-17], researches have been focusing on revealing the mechanisms underlying EMT and developing strategies to block or reverse this oncogenic process. 

NF-κB pathway is involved in immune responses, apoptosis, cell proliferation, and EMT process [18]. The regulation of EMT-related genes by NF-κB promotes cellular migration and tumor metastasis [19, 20]. Twist is a critical transcription factor for promoting metastasis via regulating the EMT pathway that can be modulated by NF-κB [21-23]. Wnt, Notch, and NF-kB network has pivotal roles in tumor microenvironment and metastasis [24, 25]. *VOPP1*, also known as EGFR-coamplified and overexpressed protein, is a critical regulator of apoptosis and growth in cancer cells that primarily acts through the NF-κB pathway [26, 27]. *VOPP1* has a dual role as either an oncogene or tumor suppressor, depending on the cellular context. *VOPP1* has been associated with increased tumor cell survival in gastric cancer [28]. Conversely, *VOPP1* suppressed cell growth and invasion in cervical cancer [29]. These findings suggest that *VOPP1* may contribute to tumor progression by modulation of signaling pathways, emphasizing its dual functionality and importance in cancer biology. Considering the role of NF-κB, WNT, and NOTCH network in tumor microenvironment and the lack of reports on the role of *VOPP1* in ovarian cancer, we investigated its role in regulation of EMT process and drug resistance via WNT and NOTCH pathways.

## MATERIALS AND METHODS

### Cell culture and transfection:

A2780 cell line was cultured in RPMI 1640 medium supplemented with 10% FBS, 100 U/mL penicillin, and 100 μg/mL streptomycin. Transfection of the A2780 cells was performed using a *VOPP1* expression vector (p3XFLAG-CMV-7.1-*VOPP1*) and a specialized nanoparticle delivery system (Fusofect, Iran). Since, p3XFLAG-CMV-7.1 had not any visual marker; we used pCDH-513b plasmid as the positive control to check the efficiency of transfection. Transfection efficiency was assessed 48 hours post-transfection using optical evaluation (Optica, Italy). Additionally, real-time PCR analysis confirmed the successful up regulation of *VOPP1* expression in transfected cells (12 fold changes).

### RNA extraction, cDNA synthesis, and real-time PCR:

RNA was extracted from both transfected and control A2780 cells using the Total RNA Extraction Kit (Parstous, Iran). cDNA synthesis was performed using Easy cDNA Ultra-TM Synthesis Kit (Parstous, Iran), and real-time PCR was conducted using the SYBR Green method (Parstous, Iran) in duplicate reactions (Light Cycler, Germany). The expression levels of *VOPP1*, EMT markers, Notch, Wnt, and ABC transporter components were assessed in transfected cells compared to non-transfected controls. All of the primer sequences were designed by AlleleID®, and then were checked by primer blast database (Table 1) [30-32]. *GAPDH* served as internal normalizers for the mRNA expression analysis. Gene expression was analyzed using the -ΔΔCT method, with changes in fluorescence intensity of >|2-fold| considered as deregulation [33, 34].

**Table 1:** Primer sequences used for comparative real-time qRT-PCR

**Genes**	**Forward**	**Reverse**
***VOPP1***	GGCTGTGGTACTTCTGGTTCCTT	GTGTAGGACACATTGAAGGCTGG
***WNT1***	GTCCTCCTAAGTCCCTTCCTATTC	CCCAACCTCATTTCCACATCATC
***β-catenin***	CAACTAAACAGGAAGGGATGGAAGG	CAGATGACGAAGAGCACAGATGG
***FZD-1***	CAAGAGAGGAGCCGAGAAAGTATG	CCAGCAGCCAAAGCAGCAG
***GSK3-B***	AGTGGTGAGAAGAAAGATGAGGTC	GTTTAATATCCCGATGGCAGATTCC
***LEF-1***	CAGCGGAGCGGAGATTACAG	GATTTCAGACTCGTTCACCAAGG
***TCF-7***	GCTGCCATCAACCAGATCCT	CCTCCTGTGGTGGATTCTTGG
***DVL***	GGTCTCCTGGCTGGTCCTG	CCTGTCTCGTTGTCCATCCC
***LRP6***	AACCTTCAAGAATACAGACAGCAC	TCTTCACATTCAGTAAACCCATCG
***TWIST1***	GGAGTCCGCAGTCTTACGAG	GTCATTGATGGCAACAATATCCACT
***VIMENTIN***	GGCTCGTCACCTTCGTGAAT	GAGAAATCCTGCTCTCCTCGC
***N-CADHERIN ***	ATGGTGTATGCCGTGAGAAG	TGTGCTTACTGAATTGTCTTGG
***FIBRONECTIN***	AGGAAGCCGAGGTTTTAACTG	AGGACGCTCATAAGTGTCACC
***SNAIL***	CTAGGCCCTGGCTGCTACAA	ACATTCGGGAGAAGGTCCGA
***SLUG***	GCCAAACTACAGCGAACTGG	TGGAATGGAGCAGCGGTAG
***E-CADHERIN***	ATTCTGATTCTGCTGCTCTTG	AGTCCTGGTCCTCTTCTCC
***OCCLUDIN***	AAGCAAGTGAAGGGATCTGC	GGGGTTATGGTCCAAAGTCA
***ZEB-2***	GGGACAGATCAGCACCAAAT	CGCAGGTGTTCTTTCAGATG
***I-CAM***	TGTGACCAGCCCAAGTTGTT	AGTCCAGTACACGGTGAGGA
***MMP-2***	GCAGGGCGGCGGTCAC	CGAAGGCAGTGGAGAGGAAGG
***MMP-3***	GCTGTATGAAGGAGAGGCTGATATAATG	GAGAAATAAATTGGTCCCTGTTGTATCC
***MMP-7***	TAAAGGCATTCAGAAACTATATGGAAAGAG	GGAGTGGAGGAACAGTGCTTATC
***MMP-9***	GACGCCGCTCACCTTCACTC	GGAACCACGACGCCCTTGC
***MMP-10***	AAGAGATGCTGTTGATTC	ATTGGTGCCTGATGC
***HES1***	GGCTAAGGTGTTTGGAGGCT	GCTGTTGCTGGTGTAGACGG
***HES5***	AAGCACAGCAAAGCCTTC	GCACCACGAGTAGCCTTC
***HEY1***	ACGGCAGGAGGGAAAGGTTAC	CTGGGAAGCGTAGTTGTTGAGATG
***HEY2***	ATGAGCATAGGATTCCGAGAGTG	GGCAGGAGGCACTTCTGAAG
***MAML1***	TCTCGCGGAACAGGAGAA	GCAGCAGAGGACCCTGTG
***DLL1***	ATAGCAACTGAGGTGTAAAATGG	CTCGGTCTGAACTCGGTTTC
***NOTCH1***	CAGAGGCGTGGCAGACTAT	CGGCACTTGTACTCCGTCAG
***NOTCH2***	CCTTGCCTGAACGATGGTC	TCTCTGCCCTGTGAATCCTG
***NOTCH3***	AGGGACGTCAGTGTGAACTC	GTCCACATCCTGCTGGCATC
***ABCC4***	GAAATTGGACTTCACGATTTAAGG	TTCCACAGTTCCTCATCCGT
***ABCG2***	TGAGGGTTTGGAACTGTGG	GATTCTGACGCACACCTGG
***GAPDH***	GGAAGGTGAAGGTCGGAGTCA	GTCATTGATGGCAACAATATCCACT

### Migration assay and Drug resistance assay:

A2780 cells were seeded in 12-well plates and allowed to reach 90–100% confluency. A scratch was created in the monolayer, and wound closure was assessed using an inverted microscope (Optica, Italy) at 24- and 48-hours post-scratch. Images were captured, and the percentage of wound closure was calculated using ImageJ software. Migration assays were performed in duplicate reactions to quantify the extent of cell migration. Statistical significance of migration differences between transfected and control A2780 cells was evaluated using the ANOVA test. The statistical analyses were conducted using the SPSS 27.0.1 software (Chicago, IL). The response of A2780 cells to Paclitaxel (PTX) (IC_50_=0.65 µM) following *VOPP1* ectopic expression was also assessed using the MTT assay. We compared cell viability between transfected and control A2780 cells by measuring the optical density (OD) of viable cells at an absorbance of 570 nm. All of the MTT tests were conducted in triplicate.

## RESULTS

**Figure 1:**
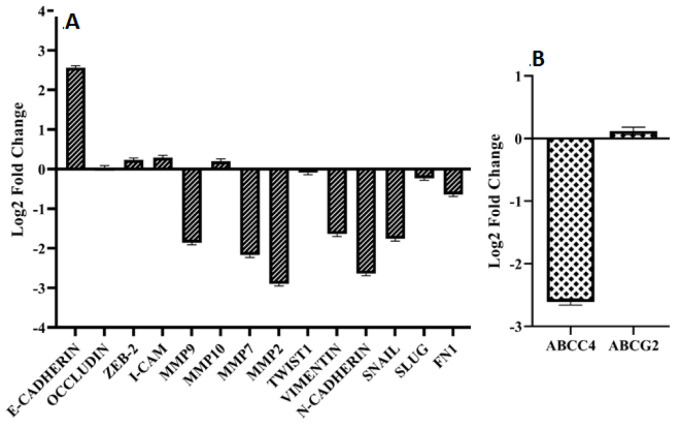
Expression level of critical genes involved in EMT process in *VOPP1* transfected compared to the non-transfected A2780 cells (A). Expression level of ABC transporters in *VOPP1* transfected compared to the non-transfected A2780 cells (B).

We assessed the role of *VOPP1 *in regulation of EMT process. There were E-cadherin up regulation (2.56 fold changes), while Vimentin, N-cadherin, and Snail down regulations (-1.64, -2.64, and -1.76 fold changes, respectively) following the *VOPP1* ectopic expression in A2780 cells. Matrix Metalloproteinases (*MMP2*, *MMP7*, and *MMP9*) were also significantly down regulated in *VOPP1*-transfected cells (-2.90, -2.17, and -1.86 fold changes, respectively). These findings suggest that *VOPP1* may act as a suppressor of EMT by down regulating transcription factors, mesenchymal markers, and MMPs, while enhancing E-cadherin expression (Fig. 1A). Tumor recurrence remains a major obstacle in cancer therapy due to drug resistance [35, 36]. Since, ABC transporters play a pivotal role in mediating drug efflux; we investigated the potential relationship between *VOPP1* and the expression of *ABCC4* and *ABCG2*. *VOPP1* down regulated the *ABCC4* (-2.61 fold changes) in A2780 cells (Fig. 1B). that results in reduced PTX efflux and reduced resistance in A2780 cells. 

**Figure 2:**
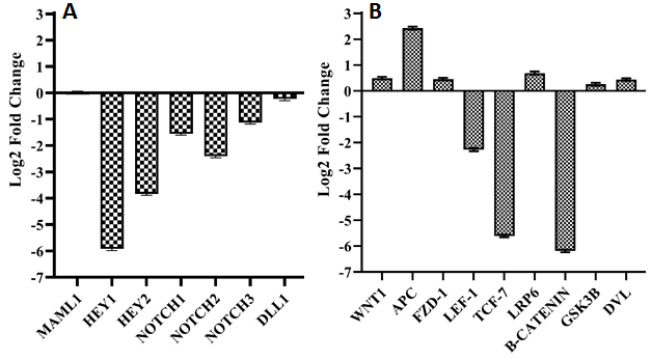
Expression level of NOTCH signaling components in *VOPP1* transfected compared to the non-transfected A2780 cells (A). Expression level of WNT signaling components in *VOPP1* transfected compared to the non-transfected A2780 cells (B).

We evaluated the potential relationship between *VOPP1* expression and NOTCH pathway through the analysis of several key components of this pathway. *VOPP1* down regulated *NOTCH1*, *NOTCH2*, and *NOTCH3* (-1.55, -2.40, and -1.13 fold changes, respectively) in A2780 cells. We also analyzed the expression of *HEY1* and *HEY2*, two major downstream target genes of the NOTCH pathway. Interestingly, *VOPP1* down regulated *HEY1* (-5.92 fold change) and *HEY2* (-3.83 fold change) in transfected cells. These results suggested that *VOPP1* might negatively regulate the NOTCH pathway in A2780 cells (Fig. 2A). We also investigated the potential link between *VOPP1* expression and the WNT pathway by analyzing the expression of several key WNT components. Notably, there was a significant up regulation of *APC* (2.43 fold change) and a marked down regulation of β-catenin (-6.19 fold change) in *VOPP1*-transfected A2780 cells. Moreover, *LEF1* and *TCF7* were significantly down regulated in *VOPP1*-transfected cells (-2.27 and -5.61 fold changes, respectively) (Fig. 2B).

**Figure 3:**
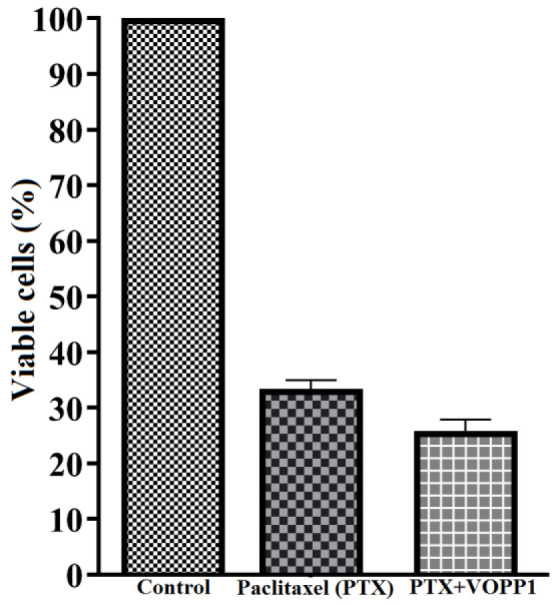
Drug resistance assay for the PTX in VOPP1 transfected cells in comparison with control A2780 cells.

**Figure 4:**
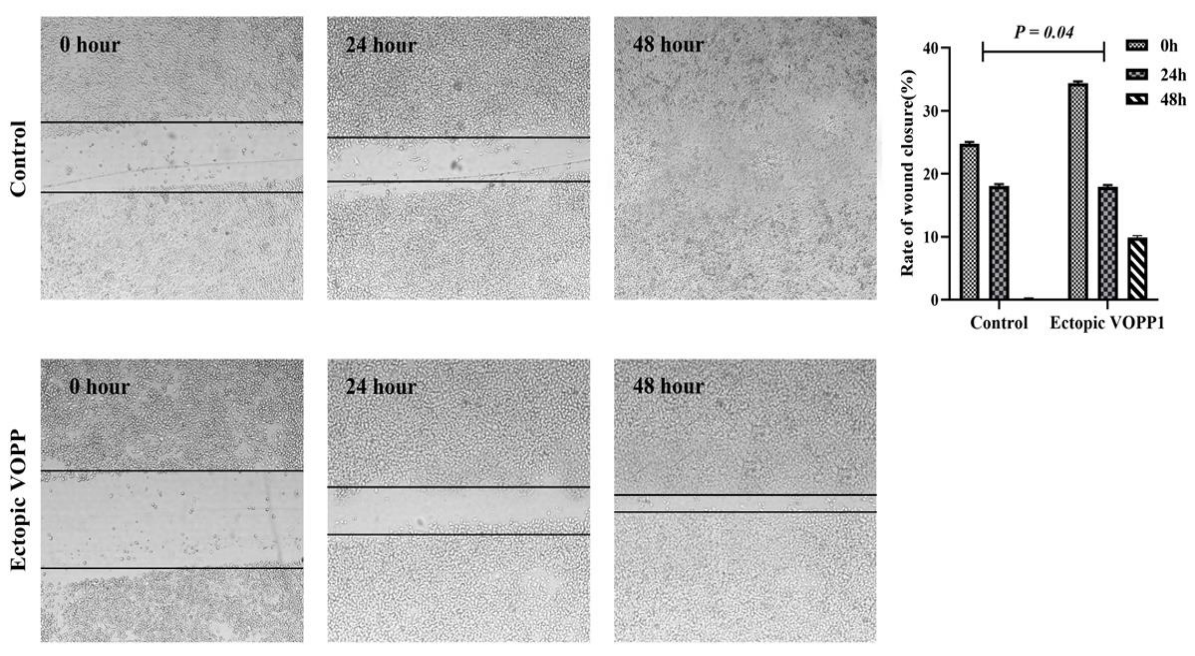
Migration assay in *VOPP1* transfected in comparison with non-transfected A2780 cells.

The effect of *VOPP1* on Paclitaxel (PTX) resistance and cell migration was evaluated in A2780 cells. *VOPP1* significantly decreased PTX resistance after 48 hours in A2780 cells (*p*<0.0001) (Fig. 3). *VOPP1* also significantly reduced A2780 cell migration at both 24 and 48 hours compared to control cells (*p*=0.04) (Fig. 4). These results indicated that *VOPP1* reduced chemo resistance and impaired cell migration in A2780 cells. 

## DISCUSSION

Crosstalk between signaling pathways has been closely associated with several hallmarks of cancer, including drug resistance, EMT process, and immunosuppression [24, 25, 37]. NF-κB signaling is widely recognized as an oncogenic pathway in tumor cells [38-41]. The interaction between NF-κB signaling and the EMT process have been extensively studied in gynecological malignancies. For example, inhibition of NF-κB signaling down regulated Twist and suppressed EMT process and cell invasion in OC [42]. PI3K/Akt axis activated NF-κB signaling that enhanced EMT process and ovarian tumor cell invasion [43]. 

It has also been demonstrated that *SOS1* can activate NF-κB mediated EMT process in an Akt-independent manner in ovarian cancer [44]. *VOPP1* is a critical regulator of cell survival through activation of the NF-κB pathway [45, 46]. *VOPP1* has dual roles as a tumor suppressor or oncogene. Reduced expression of *VOPP1* has been shown to enhance resistance toward BET inhibitors in non-small cell lung cancer (NSCLC) cells [47]. Inhibition of *HOTAIR* reduced 5-FU resistance in colorectal cancer (CRC) via *miR-218/VOPP1* axis [48]. *VOPP1* facilitates the localization of *WWOX* protein within lysosomes, preventing its interaction with *p73α*, thereby suppressing apoptosis and promoting tumor progression [49]. *VOPP1* also exerts its regulatory functions by modulating the lncRNA-miR-mRNA axes in tumor cells [28, 50]. Thus, in this study we investigated the role of *VOPP1* in ovarian tumor progression. Regarding the key role of WNT and NOTCH pathways in EMT process and drug response of tumor cells, we assessed for a probable interaction between the *VOPP1* and these signaling pathways in ovarian tumor cells. 

NF-κB signaling has been shown to suppress the Wnt/β-catenin pathway indirectly, by regulating the expression of NF-κB target genes, including *LZTS2* and *SMURF*, or directly, by interfering with the assembly of the β-catenin/TCF/p300 transcriptional complex [51]. We also observed that *VOPP1* up regulated *APC* while down regulated β-catenin in ovarian tumor cells. This suggests that *VOPP1* may regulate the stability of β-catenin by modulating the destruction complex. In addition, *LEF1* and *TCF7* were significantly suppressed in *VOPP1*-transfected cells. This indicates that *VOPP1* as a part of NF-κB pathway can modulate WNT pathway via the downregulation of β-catenin and TCF/LEF complex in ovarian tumor cells. 

Notch and NF-kB are coactivated in several cancers including pancreatic, cervical and breast cancer [52, 53]. However, there is not any report about the probable interaction between NF-kB and NOTCH pathways during OC progression. Therefore, in the present study we assessed the role of *VOPP1* in OC progression via interaction with NOTCH pathway. *VOPP1* inhibited the NOTCH pathway in ovarian tumor cells. EMT process was demonstrated to be regulated by activation of Wnt/β-catenin pathway in OC [54-56]. Notch-induced EMT was shown to increase cell migration and sphere formation of ovarian cancer cells [57-59]. Therefore, WNT and Notch are pivotal regulators of EMT process in ovarian cancer and we hypothesized that *VOPP1* can suppress the EMT process through regulation of WNT and Notch pathways. *VOPP1* significantly reduced EMT and cell migration in ovarian tumor cells. We also observed a marked down regulation of *MMP2*, *MMP7*, and *MMP9* as key factors in cell migration in *VOPP1*-transfected cells. Therefore, *VOPP1* can be a critical regulator EMT process in OC cells through inhibition of WNT and Notch pathways. Chemo resistance can be regulated by a variety of molecular and cellular mechanisms, such as the ATP-binding cassette (ABC) transporters and EMT process [14, 60, 61]. *ABCG2* up regulation was shown to develop cisplatin and paclitaxel resistances in ovarian tumor cells [62-64]. Our results revealed a significant down regulation of *ABCC4* expression in *VOPP1*-transfected cells. *VOPP1* reduced PTX resistance in ovarian tumor cells by regulation of *ABCC4*. 

Our study highlighted the pivotal role of *VOPP1* as a negative regulator of WNT/β-catenin and NOTCH pathways in ovarian tumor cells. *VOPP1* suppressed the EMT process, PTX resistance, and OC cell migration by inhibition of WNT and NOTCH pathways. Therefore, *VOPP1* can be considered as a potential target for overcoming drug resistance and inhibiting tumor metastasis in OC patients following further animal studies and clinical trials. 
